# Rho, ROCK and actomyosin contractility in metastasis as drug targets

**DOI:** 10.12688/f1000research.7909.1

**Published:** 2016-04-29

**Authors:** Irene Rodriguez-Hernandez, Gaia Cantelli, Fanshawe Bruce, Victoria Sanz-Moreno

**Affiliations:** 1 Tumour Plasticity Laboratory, Randall Division of Cell and Molecular Biophysics, Guy’s Campus, King’s College London, London, SE1 1UL, UK; 2 Department of Imaging Chemistry and Biology, Division of Imaging Sciences and Biomedical Engineering, St. Thomas Hospital, King’s College London, London, SE1 7EH, UK

**Keywords:** Rho-ROCK, actomyosin, metastasis, Rho

## Abstract

Metastasis is the spread of cancer cells around the body and the cause of the majority of cancer deaths. Metastasis is a very complex process in which cancer cells need to dramatically modify their cytoskeleton and cope with different environments to successfully colonize a secondary organ. In this review, we discuss recent findings pointing at Rho-ROCK or actomyosin force (or both) as major drivers of many of the steps required for metastatic success. We propose that these are important drug targets that need to be considered in the clinic to palliate metastatic disease.

## Introduction

Metastatic disease is still largely incurable because of its systemic distribution and resistance to current therapies, and it is the cause of more than 90% of cancer-related deaths ^1, 2^. In spite of its clinical importance, the underlying cellular and molecular mechanisms of cancer metastasis are only partially understood ^3^. Thus, improved knowledge of how cancer cells acquire metastatic traits is necessary to unravel novel drug targets and prognostic markers of distant relapse.

Metastasis is a complex multi-stage process by which cancer cells disseminate from primary tumors, survive in distant sites and eventually grow as secondary tumors ^3^. The main events of the metastatic cascade involve loss of cell-cell contacts, cancer cell migration, local invasion of the surrounding extracellular matrix (ECM), interactions with stroma, intravasation and transit into blood or lymphatic vessels, arrest at secondary sites, extravasation, survival and colonization of distant sites ^4^. Genetic alterations and deregulation of critical oncogenic signaling pathways affecting survival, proliferation, apoptosis, and cell motility, regulate many of these complex metastatic events ^3, 5^. In addition, the interaction with the tumor microenvironment such as ECM, growth-supportive stromal cells, inflammatory cells and endothelial cells strongly impacts the metastatic capabilities of cancer cells ^6, 7^.

Many signaling pathways have been reported to have an impact on metastasis and have been the focus of excellent reviews ^8– 15^. In the present review, we will focus on Rho-ROCK signaling and actomyosin contractility, key regulators of several main steps in metastasis. Rho-ROCK, through its actions on cytoskeletal dynamics and through regulation of critical signaling pathways, controls several cellular processes important for metastasis such as cell migration, local invasion, survival at the secondary site, and tumor outgrowth ^16– 18^.

## Rho GTPases and metastasis

The Rho family of small GTPases plays crucial roles in the regulation of the actin cytoskeleton, cell polarity, cell migration, cell proliferation, invasion, and metastasis ^19^. Rho GTPases act as molecular switches cycling between a guanosine triphosphate (GTP)-bound active state and guanosine diphosphate (GDP)-bound inactive state to translate extracellular signals into different cellular responses ^19^. Their activity is controlled by guanine nucleotide exchange factors (GEFs) and GTPase-activating proteins (GAPs) ^18^. The best studied and most conserved Rho family members across eukaryotic species are Ras-related C3 botulinum toxin substrate 1 (Rac1), cell division control protein 42 homolog (Cdc42), and Ras homolog gene family member A (RhoA) ^18^. Rac1 stimulates lamellipodia formation ^20^, whereas RhoA regulates the formation of stress fibers or favors amoeboid migration depending on the cellular context and the properties of the matrix. RhoA bound to GTP leads to activation of its effectors Rho-associated protein kinases (ROCK1 and ROCK2) ^21– 23^. ROCK1/2 serine/threonine kinases promote actomyosin contractile force generation by decreasing myosin phosphatase activity and thereby increasing phosphorylation of myosin light chain 2 (MLC2) ^24^. On the other hand, Cdc42 induces filopodia formation ^25^, but Cdc42 signaling can also generate actomyosin contraction through p21 protein (Cdc42/Rac)-activated kinase 2 (PAK2) and myotonic dystrophy kinase-related Cdc42-binding kinase (MRCK) kinases ^26, 27^. Deregulation of the Rho-ROCK signaling pathway has been found in a variety of cancer types and in several cases correlates with disease progression ^28– 30^ ( Table 1). Furthermore, inhibition of ROCK signaling could suppress migration and invasion *in vitro* and impair the metastatic process *in vivo*, suggesting that ROCK inhibitors might be potential anti-metastatic agents ^30– 32^.

**Table 1.** Rho, ROCK or actomyosin contractility are implicated in all stages of the metastatic cascade and in major cancer types. Shown are examples in the literature of where different stages of the metastatic cascade have been shown to be influenced by Rho-ROCK and/or actomyosin contractility signalling. (SCC= Squamous cell carcinoma)

Cancer type	Step of metastatic dissemination	Reference
Breast, colon	Local invasion and migration	40, 46, 52, 68
Breast	Intravasation	40, 66, 71
Breast	Survival in circulation, adhesion to vessels, early lung colonization	92
Oesophageal	Invasion and survival in circulation	104
Lung	Transendothelial migration	81
Prostate	Transendothelial migration	75
SCC	Fibroblast mediated invasion and migration	42
Melanoma	Local invasion and migration	23, 32, 45, 46, 49, 105
Melanoma	Intravasation, extravasation, survival in circulation, adhesion to vessels, early lung colonization	23, 32, 48, 54, 58, 92, 95– 97, 105

## Rho/ROCK signaling and actomyosin contractility in early dissemination

The ability of cancer cells to migrate into and invade surrounding tissue is a critical step in the metastatic cascade, which requires increased cell motility driven by altered cytoskeletal organization and contacts with the ECM and the stroma ^33^. Cancer cells can move either collectively or as individual cells ^34, 35^. The majority of tumors originate from epithelial tissues, and epithelial cancer cells that leave the primary tumor undergo a complex program called epithelial-mesenchymal transition (EMT). Incomplete or partial EMT allows collective migration in which cells can maintain cell-cell adhesions and migrate collectively in a coordinated manner as strands, sheets, or cell clusters. On the other hand, complete EMT is associated with the loss of cell-cell adhesions in favor of cell-ECM interactions and the concomitant acquisition of individual migratory characteristics ^36, 37^. After undergoing EMT, individual cancer cells can engage into elongated mesenchymal or rounded amoeboid modes of movement, distinguished by their different usage of signaling pathways. Mixed mesenchymal and amoeboid phenotypes have also been identified ^38, 39^. Individual cell migration seems to be required for blood-borne metastasis ^40^.

### Rho/ROCK signaling and actomyosin contractility in cancer cells

Actomyosin contractility driven by Rho or ROCK signaling is key in controlling tumor dissemination, as all forms of cell migration require a certain degree of actomyosin force ^34, 41^. During collective cell migration, actomyosin contractility is high around the edges of groups of invading cancer cells, which generates pulling forces between the substrate and the follower cells, together with a prominent actomyosin ring at lateral regions of the groups to maintain coupling between cells and collective forward movement ^42, 43^. On the other hand, in individual migration, the contractile cortex is crucially important for amoeboid to intermediate forms of movement, and some degree of contractility is also required to retract protrusions in mesenchymal migration ^39, 44– 46^. The mesenchymal mode of movement is characterized by an elongated, spindle-like shape, high levels of adhesion, and Rac-dependent adhesive actin-rich protrusions ^23, 46, 47^. On the other side of the spectrum, in amoeboid migration, cancer cells adopt a rounded or irregular morphology with blebs as functional protrusions. Amoeboid motility is promoted by high levels of RhoA/Ras homolog gene family member C (RhoC) or ROCK-driven actomyosin contractility and requires lower levels of adhesion that allow higher speeds of movement ^46– 50^.

**Figure 1.**
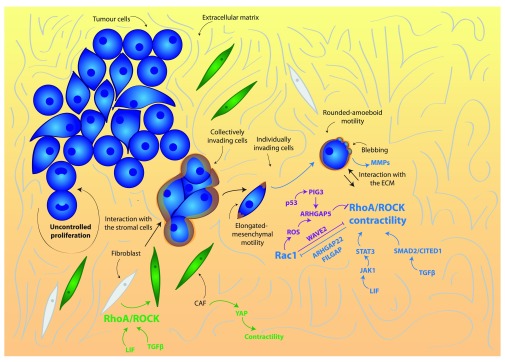
Rho/ROCK and actomyosin contractility in early dissemination. ROCK-driven actomyosin contractility is stimulated by extracellular signals such as leukemia inhibitory factor (LIF) and transforming growth factor beta (TGFβ) to promote rounded amoeboid cancer cell motility. Rounded amoeboid cells display blebbing as well as high levels of actomyosin contractility and a rounded morphology. They interact with the extracellular matrix (ECM) by physically deforming it and by secreting metalloproteases (MMPs). In the stroma, ROCK-driven actomyosin contractility promotes the transformation of fibroblasts into cancer-associated fibroblasts (CAFs), driven by Yes-associated protein (YAP) as well as by extracellular factors. Blue indicates positive regulators of contractility, purple indicates negative regulators of contractility and orange lines indicate actomyosin contractility. Abbreviations: CAF, carcinoma-associated fibroblasts; CITED1, Cbp/P300-interacting transactivator with Glu/Asp-rich carboxy-terminal domain, 1; FilGAP, filamin-A-associated Rho GTPase activation protein; JAK, Janus kinase; RhoA, Ras homolog gene family member A; ROCK, Rho-associated protein kinase; SMAD2, Sma- and Mad-related protein 2; STAT3, signal transducer and activator of transcription 3.

Cancer cell migration is a dynamic process, and individual cancer cells can switch between modes of movement to adapt to the changing microenvironment and facilitate tumor dissemination. Different cues will favor either a mesenchymal-amoeboid transition (MAT) or an amoeboid-mesenchymal transition (AMT) ^23, 45, 49, 51, 52^. Their core regulatory network is the mutually inhibitory circuit between Rac1 and Rho GTPase signaling in migrating cells ( Figure 1). Higher Rac1 activity promotes cell elongation and permits long actin-rich protrusions characteristic of mesenchymal migration. Moreover, active Rac1 negatively regulates Rho or ROCK signaling and suppresses amoeboid movement. On the other hand, active Rho or ROCK supports bleb-based amoeboid migration ^23, 45, 49, 51, 52^ and limits excessive Rac1-dependent adhesion via regulation of the Rac GAPs ARHGAP22 and filamin-A-associated Rho GTPase activation protein (FilGAP) ^23, 53^. Furthermore, cancer cells control amoeboid migration at the transcriptional level under circumstances in which matrix compliance allows sustained actomyosin contractility ( Figure 1). Different chemical cues have been shown to control this process. For instance, amoeboid melanoma cells support contractility, establishing a positive feedback loop with the cytokines leukemia inhibitory factor (LIF)/IL6 and the Janus kinase (JAK)/signal transducer and activator of transcription (STAT) pathway to maintain Rho-ROCK activity ^49^. As a result of high STAT3 activity, very contractile cells secrete different factors, including matrix metalloprotease 9 (MMP-9). MMP-9 promotes the generation of actomyosin contractile force and bleb-driven migration through a positive feedback loop via CD44 binding and increased MLC2 phosphorylation to sustain amoeboid invasion ^48^. Moreover, amoeboid contractile cells secrete high levels of transforming growth factor beta (TGFβ), and downstream of it a Sma- and Mad-related protein 2 (SMAD2)-Cbp/P300-interacting transactivator with Glu/Asp-rich carboxy-terminal domain (CITED1) transcriptional network sustains actomyosin contractility ^54^. In addition, the physical properties of the matrix play an important role in establishing a balance between actomyosin levels and adhesion to regulate optimal migration efficiency ^34, 39, 47, 55, 56^. Increased ECM density results in increased matrix stiffness, in which cells sense and respond by increasing Rho-mediated actomyosin contractility ^57^. Furthermore, slow mesenchymal cells can switch to fast amoeboid migrating modes under conditions of low adhesiveness and high physical confinement ^47, 56^.

The ability to switch between different modes of migration is an important factor for metastatic dissemination, as cancer cells have to migrate through a range of ECMs to escape the primary tumor and spread to distant organs. Therefore, anti-metastatic treatments should target the ability of tumor cells to cope with such variability. Recently, it has been described that potent ROCK inhibitors are able to strongly inhibit actomyosin contractility and collapse the actomyosin cytoskeleton, blocking both mesenchymal and amoeboid modes of movement ^32^.

Intra-vital imaging studies have shown that bleb-driven highly contractile amoeboid migration is favored in the invasive fronts of melanomas and breast cancers ^23, 29, 45, 48, 49, 58^. Furthermore, in these studies, it has been shown that treatment with ROCK inhibitors or actomyosin perturbations (or both) is able to decrease tumor cell motility *in vivo* ^23, 29, 32, 45, 49, 58^. Hence, ROCK inhibition could effectively impair local invasion and dissemination of cancer cells ( Figure 1).

### Rho/ROCK signaling and actomyosin contractility in the stroma

Within the tumor, a variety of non-cancer stromal cells interact with the cancer cells promoting tumorigenesis ^7^. Actomyosin contractility not only is fundamental for cancer cell migration and invasion but also is crucial for maintenance of the carcinoma-associated fibroblasts (CAFs) phenotype, an important stromal component in the tumor microenvironment ^7^. Actomyosin contractility activated by ROCK signaling and the LIF/JAK/STAT pathway is crucial for CAF-dependent pro-invasive physical remodeling of the ECM favoring tumor aggressiveness and dissemination ^42, 49, 59, 60^. Additionally, actomyosin contractility, Src function, and matrix stiffening induced by TGFβ, are required for Yes-associated protein (YAP) activation in CAFs to promote ECM remodeling and cancer cell invasion, and to generate a positive feedback loop that helps to maintain the CAF phenotype ^61^ ( Figure 1). Moreover, contractility in CAFs has been shown to modulate EMT and metastasis-initiating cell properties in breast cancer models ^62^.

Therefore, some degree of actomyosin contractility is essential for both cancer cells and stroma for efficient cell movement in the initial steps of the metastatic cascade ^34, 41, 49, 59, 61^, and some factors such as TGFβ and LIF can stimulate contractility both in cancer cells and in fibroblasts.

## Rho/ROCK signaling and actomyosin contractility in transendothelial migration

After local invasion within the primary tumor microenvironment, cancer cells need to spread throughout the body and colonize new organs to form metastases. They do so by exploiting the vascular and lymphatic systems. The process through which cancer cells enter and exit vessels crossing the endothelial layer is known as transendothelial migration, which is extremely complex and involves the interaction with several different cell types, such as platelets, immune cells and endothelial cells, and the activation of a variety of signaling pathways ^63^. These events are in some cases similar to those occurring during inflammation or infection, when immune cells need to enter and exit vessels. In fact, parallels between cancer cell and immune cell migration allow for interesting speculation in areas of cancer cell dissemination that are still not fully understood.

**Figure 2.**
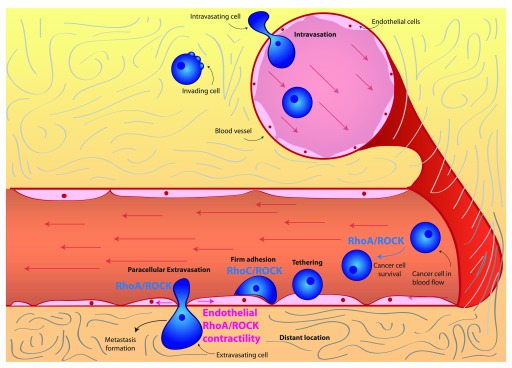
Rho/ROCK and actomyosin contractility in intravasation and extravasation. RhoC/ROCK signaling promotes survival of cancer cells in the blood flow as well as adhesion to the endothelium and extravasation. ROCK-driven actomyosin contractility within endothelial cells can be stimulated by secreted factors and is essential for cancer cell extravasation. Abbreviations: RhoC, Ras homolog gene family member C; ROCK, Rho-associated protein kinase.

***Intravasation*.** The first step in this metastatic cascade is intravasation, the entry of tumor cells into blood vessels. Intravasation depends on the weakening of cell-cell junctions between endothelial cells, which allows cancer cells to squeeze in between adjacent endothelial cells and enter the vessel lumen ^63^. From a molecular perspective, not as much is known about intravasation compared with other steps in the metastatic cascade as this is an experimentally challenging step to study ^64, 65^. In fact, intravasation is dependent on the ability of cancer cells to invade towards blood vessels, so it is difficult to distinguish between genes involved in invasion and intravasation ^63^. RhoA signaling has been linked to the process of intravasation ^66^ ( Figure 2). Specifically, RhoA activity in cancer cells is thought to be stimulated by macrophage contact and leads to the formation of invadopodia. Invadopodia are instrumental in the degradation and eventual breakdown of the matrix barrier, which allows for tumor cell intravasation. Furthermore, highly contractile, rounded amoeboid melanoma cells have been shown to intravasate more efficiently than low-contractility elongated cells *in vivo* ^67, 68^. Once in the bloodstream, cells are transported throughout the body by the blood flow ( Figure 2).

***Extravasation.*** Eventually, cancer cells flowing through the bloodstream need to exit blood vessels to form secondary tumors. This process is known as extravasation and entails several sequential steps. First of all, cancer cells form loose adhesions to the vascular endothelium, which is known as tethering. These loose adhesions then are tightened to form firm adhesions: firmly adhering cells then can cross the endothelial barrier and extravasate ^63^.

The best-studied mechanism for extravasation is known as paracellular extravasation, during which cancer cells exit the vessel by squeezing in between endothelial cells. An alternative mechanism for cancer cell extravasation is transcellular extravasation, where tumor cells exit the vessel by going through endothelial cells ^63, 69^. Transcellular extravasation has been observed in immune cells ^70^ and has also been identified in cancer cells, where it probably plays a role in some cases ^71^.

### Rho/ROCK signaling and actomyosin contractility in cancer cells

Rho or ROCK-driven actomyosin contractility within extravasating cells has been shown to play an important role. For instance, in monocytes, RhoA activity has been shown to be necessary for tail retraction during extravasation ^72^. In the context of transcellular extravasation, monocytes can rely on RhoA and ROCK signaling ^73, 74^.

On the other hand, in prostate cancer cells, it is RhoC and ROCK signaling that is essential for interaction with endothelial cells, promoting adhesion and paracellular extravasation ^75^. As a result of its role in promoting extravasation, RhoC signaling is a key driver of tumor dissemination and metastasis ^75^, in part explaining how *RhoC* was one of the first genes identified as a metastasis driver ^76^. Furthermore, RhoA and RhoC have been shown to drive adhesion to the endothelium and transendothelial migration in breast and prostate cancer cells ^77, 78^. Consequently, rounded-amoeboid cancer cells with high levels of RhoA or ROCK-driven actomyosin contractility are more efficient during transendothelial migration than elongated cells both *in vitro* and *in vivo* ^67, 68, 79^. Additional evidence supporting the importance of RhoA-driven contractility in transendothelial migration comes from studies examining the role of RhoA regulators. For instance, FilGAP, a Rac GAP, promotes RhoA signaling and rounded-amoeboid motility by suppressing Rac, and as a consequence it enhances *in vivo* extravasation of breast cancer cells ^53^. Conversely, the RhoA GAP ARHGAP7 has been shown to be a negative regulator of transendothelial migration in thymic lymphoma ^80^.

Cancer cells that successfully extravasate need to cross the vascular basement membrane that surrounds the vessel ^63^. Since actomyosin contractility has been shown to promote the secretion of proteases in rounded amoeboid cells ^48^, it is tempting to speculate that highly contractile extravasating cells could have an advantage when crossing the vascular basement membrane.

### Rho/ROCK signaling and actomyosin contractility in endothelial cells

In order for paracellular extravasation to occur, cancer cells need to weaken cell-cell junctions within the endothelium. This can be mediated by regulating Rho or ROCK signaling and actomyosin contractility within the endothelial cells themselves ( Figure 2). Lung cancer cells have been shown to induce adherens junction disassembly by stimulating actomyosin contractility through Rho/ROCK in endothelial cells ^81^. Furthermore, thrombin stimulation of endothelial cells has been shown to induce ROCK activity and subsequently lead to cytoskeletal remodeling, junction disruption, and endothelial permeability ^82, 83^. Tumor-derived thrombin induces endothelial gap formation and transendothelial migration ^84^. Furthermore, cancer cells have been shown to use thrombin within blood vessels in order to promote metastasis ^85^. This prompts the speculation that actomyosin contraction in endothelial cells could be controlled by thrombin produced by cancer cells.

As well as leading to junction disassembly, actomyosin contractility in endothelial cells allows for endothelial cell retraction ^86, 87^, which increases endothelial permeability. Moreover, ROCK-driven actomyosin contractility in endothelial cells has been shown to prevent endothelial cell re-spreading downstream of ephrin-B signaling, which maintains increased endothelial permeability ^88^. Conversely, ROCK inhibition has been shown to decrease endothelial permeability after hemorrhage ^89, 90^. Although these studies have not been conducted in cancer models, ROCK activity in endothelial cells could be similarly regulated while in contact with disseminating cancer cells.

In brief, we speculate that the ability of cancer cells to form secondary tumors is to a certain extent dependent on their ability to manipulate the cytoskeleton of endothelial cells; thus, increasing endothelial permeability could be a crucial step to promote extravasation. More work is needed to validate the roles of Rho/ROCK or actomyosin contractility (or both) in tumor cells during both cancer intravasation and extravasation.

## Rho/ROCK signaling and actomyosin contractility in metastatic colonization

Following extravasation at secondary sites, cancer cells that survive can form micro-metastasis and colonize new sites. In order for this colonization to take place, cancer cells must be able to adhere to endothelial cells, extravasate, survive and proliferate at the secondary site. The first few hours of colonization are crucial in determining the success of this process, as cells will undergo apoptosis if they do not adhere to their new niche. Furthermore, once established, cells must be able to evade the immune response in order to survive ^91^. Although we have discussed that Rho/ROCK signaling is important for early dissemination, there is also evidence to suggest that Rho/ROCK signaling, actomyosin contractility or its regulators, or a combination of these are important for efficient colonization at secondary sites.

*In vivo* studies where cancer cells are injected intravenously (i.e., experimental metastasis assays) show that high levels of actomyosin contractility play a role in seeding of and colonizing the lung. For instance, cells selected for efficient colonization in the lung such as the highly metastatic A375M2 melanoma cell line have higher levels of RhoC ^76^, RhoA ^23^ and phosphorylated MLC2 ^48^ when compared with low metastatic A375P melanoma cells.

**Figure 3.**
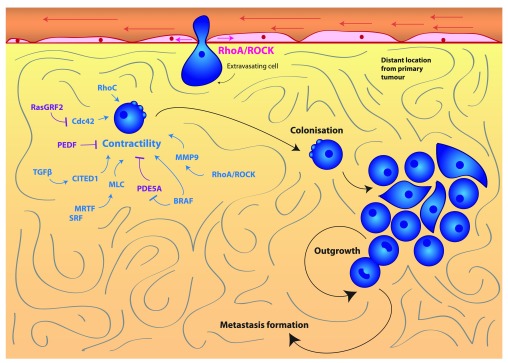
Rho/ROCK and actomyosin contractility in colonization and metastasis. Actomyosin contractility promotes cancer cell colonization and outgrowth at a secondary site to form metastases. Contractility is under the control of a wide variety of pathways, including SRF/MRTF, TGFβ-SMAD-CITED1, MMP-9, BRAF-V600E and Cdc42 signaling. Blue indicates positive regulators of contractility, and purple indicates negative regulators of contractility. Abbreviations: CITED1, Cbp/P300-interacting transactivator with Glu/Asp-rich carboxy-terminal domain, 1; Cdc42, cell division control protein 42 homolog; MLC2, myosin light chain 2; MMP, matrix metallopeptidase; MRTF, myocardin-related transcription factors; PDE5A, phosphodiesterase 5A; PEDF, pigment epithelium-derived factor; RasGRF2, Ras protein-specific guanine nucleotide-releasing factor 2; ROCK, Rho-associated protein kinase; SMAD, Sma- and Mad-related protein; SRF, serum response factor; TGFβ, transforming growth factor beta.

Several studies have confirmed the importance of the initial hours in seeding during colonization. For example, serum response factor (SRF) co-activators myocardin-related transcription factors (MRTFs) are able to control the expression of MLC2 ^92^ ( Figure 3). MRTF and SRF are both important for early stages of lung colonization in breast cancer and melanoma ^92^. Furthermore, depletion of MLC2 itself has also been shown to reduce lung colonization ^92^. Conversely, enhanced actomyosin contractility favors colonization: for example, depletion of the actomyosin contractility suppressors Rac1 and its GEF dedicator of cytokinesis 3 (DOCK3) favors early lung colonization ^23^. In melanoma, pigment epithelium-derived factor (PEDF) reduces lung colonization and suppresses lung tumor outgrowth ^93, 94^. PEDF is a negative regulator of Rho-ROCK signaling through supporting DOCK3-Rac1 activity ^95^ ( Figure 3). Furthermore, oncogenic BRAF suppresses phosphodiesterase 5A (PDE5A), which in turn inhibits actomyosin contractility ^96^ ( Figure 3). Therefore, re-expression of PDE5A reduces the ability of melanoma cells to colonize the lung and prevents short-term survival and long-term cancer growth in the lung ^96^.

As mentioned earlier, Cdc42 can also promote actomyosin contractility in cancer cells ^26^. Further evidence of the importance of actomyosin contractility in early colonization has been shown by experiments in which loss of Ras protein-specific guanine nucleotide-releasing factor 2 (RasGRF2), an inhibitor of Cdc42 ^97^, enhanced colonization of the lungs in a Rac-independent manner. This was associated with higher actomyosin contractility levels ^97^ ( Figure 3).

TGFβ signaling plays an important role in promoting cancer cell colonization ^40, 54, 98^ ( Figure 3). We recently found that TGFβ increases actomyosin contractility in melanoma cells ^54^. While TGFβ is known to promote EMT ^99^ in epithelial cancers, in melanoma TGFβ signals through SMAD2 and the adaptor CITED1 to support contractile amoeboid migration ^54^. TGFβ no longer sustains lung colonization in melanoma cells if the SMAD2-CITED1 axis is not functional ^54^, which serves to highlight the multiple levels in which actomyosin contractility promotes colonization.

Furthermore, ROCK regulates expression of several MMPs, including MMP-9, which promote early stages of lung colonization ^48^ ( Figure 3). While MMPs exert their catalytic function in degradation of the ECM during local invasion, the non-catalytic roles of MMP-9 could promote the survival of cancer cells at the metastatic secondary sites. For example, it has been shown that non-catalytic functions of MMP-9 regulate STAT3 functions to drive survival in B-cell chronic lymphocytic leukemia (B-CLL) cells ^100^.

From these results, it is clear that positive and negative regulators of Rho/ROCK signaling or actomyosin contractility (or both) are critical for cancer cells to efficiently colonize the metastatic sites in experimental metastasis models.

We have highlighted the crucial role that Rho/ROCK signaling or actomyosin contractility play in dissemination and metastatic colonization using a range of experimental cancer models. A highly contractile phenotype is clearly critical for effective cancer colonization, ultimately supporting the idea of developing drugs to inhibit actomyosin contractility. *In vivo* validation of the role of Rho/ROCK signaling or actomyosin contractility (or both) in metastasis is important to qualify these signaling modules as potential drug targets. Experimental metastasis models are insightful for understanding the processes of extravasation and colonization to the lungs, but recapitulation of the entire metastatic cascade, including local invasion, dissemination and intravasation, requires the use of spontaneous metastasis models ^101^. Indeed, it has recently been shown that a new class of ROCK inhibitors has the ability to prevent both experimental and spontaneous metastases formation ^32^. It will be of great importance to combine these mouse models with non-invasive cell-tracking techniques ^102, 103^ to understand the entire process and how early Rho/ROCK signaling should be targeted in order to effectively block the metastatic cascade.

## Abbreviations

CAF, carcinoma-associated fibroblasts; CITED1, Cbp/P300-interacting transactivator with Glu/Asp-rich carboxy-terminal domain, 1; Cdc42, cell division control protein 42 homolog; DOCK3, dedicator of cytokinesis 3; ECM, extracellular matrix; EMT, epithelial-mesenchymal transition; FilGAP, filamin-A-associated Rho GTPase activation protein; GEF, guanine nucleotide exchange factor; GAP, GTPase activation protein; GTP, guanosine triphosphate; JAK, Janus kinase; LIF, leukemia inhibitory factor; MLC2, myosin light chain 2; MMP, matrix metallopeptidase; MRTF, myocardin-related transcription factors; PDE5A, phosphodiesterase 5A; PEDF, pigment epithelium-derived factor; Rac1, Ras-related C3 botulinum toxin substrate 1; RasGRF2, Ras protein-specific guanine nucleotide-releasing factor 2; RhoA, Ras homolog gene family member A; RhoC, Ras homolog gene family member C; ROCK, Rho-associated protein kinase; SMAD2, Sma- and Mad-related protein 2; SRF, serum response factor; STAT, signal transducer and activator of transcription; TGFβ, transforming growth factor beta.
